# Fetal Programming of Adult Glucose Homeostasis in Mice

**DOI:** 10.1371/journal.pone.0007281

**Published:** 2009-09-30

**Authors:** Christopher R. Cederroth, Serge Nef

**Affiliations:** Department of Genetic Medicine and Development, University of Geneva Medical School, Geneva, Switzerland; Cincinnati Children's Research Foundation, United States of America

## Abstract

**Background:**

Emerging evidence suggests that dietary soy and phytoestrogens can have beneficial effects on lipid and glucose metabolism. We have previously shown that male mice fed from conception to adulthood with a high soy-containing diet had reduced body weight, adiposity and a decrease in glucose intolerance, an early marker of insulin resistance and diabetes.

**Objectives:**

The purpose of this study was to identify the precise periods of exposure during which phytoestrogens and dietary soy improve lipid and glucose metabolism. Since intrauterine position (IUP) has been shown to alter sensitivity to endocrine disruptors, we also investigated whether the combination of IUP and fetal exposure to dietary phytoestrogens could potentially affect adult metabolic parameters.

**Methods:**

Male outbred mice (CD-1) were allowed *ad libitum* access to either a high soy-containing diet or a soy-free diet either during gestation, lactation or after weaning. Adiposity and bone mass density was assessed by dual x-ray absorptiometry. Glucose tolerance was assessed by a glucose tolerance test. Blood pressure was examined by the tail-cuff system.

**Results:**

Here we show that metabolic improvements are dependent on precise windows of exposure during life. The beneficial effects of dietary soy and phytoestrogens on adiposity were apparent only in animals fed post-natally, while the improvements in glucose tolerance are restricted to animals with fetal exposure to soy. Interestingly, we observed that IUP influenced adult glucose tolerance, but not adiposity. Similar IUP trends were observed for other estrogen-related metabolic parameters such as blood pressure and bone mass density.

**Conclusion:**

Our results suggest that IUP and fetal exposure to estrogenic environmental disrupting compounds, such as dietary phytoestrogens, could alter metabolic and cardiovascular parameters in adult individuals independently of adipose gain.

## Introduction

During the perinatal period, a mammal is highly susceptible to endocrine disruption. This can permanently alter essential cellular functions, potentially leading to adult disorders such as infertility, metabolic disorders and cancer. Although the fetal origin of some reproductive disorders related to the exposure to man-made or environmental endocrine disrupting chemicals (EDCs) is rather well established, there are emerging data that suggest that these compounds may act as “obesogens” [1].

Most EDCs are characterized by their capacity to mimic estrogen actions. In humans, concerns about the fetal susceptibility to exogenous estrogens (xenoestrogens) originated from the findings that children from mothers who had been treated with diethylstilbestrol (DES), a potent synthetic estrogen used during pregnancy for the prevention of miscarriages, had higher risk of developing cancer in reproductive organs [2], [3], [4]. Recent data show that postnatal exposure to DES triggers obesity later in life, suggesting that environmental compounds with estrogenic activity may act as obesogens, and contribute to the current obesity pandemic [5], [6]. In addition, plant estrogens (phytoestrogens) such as those found in soybean, modulate energy expenditure, adiposity and glucose tolerance in rodents (for review see [7]). Evaluating the extent to which environmental compounds positively or negatively modulate metabolic features will significantly further our understanding of the non-genetic origin of metabolic diseases.

The most important source of human exposure to phytoestrogens is the consumption of soy and soy-derived products, which contain isoflavones - a class of phytoestrogens. Phytoestrogens have the capacity of binding to both estrogen receptor (ER) α and β, and to mimic estrogenic actions [8], [9]. The conformation of the receptor, and by inference its transcriptional response, is dependent on the ligand, and in turn enables the recruitment of various coregulators (coactivators or corepressors). As a consequence, the transcriptional landscape of estrogen receptors is highly dependant on the ligand, its concentration, and on the cellular context (cytoplasmic and nuclear environment).

Since both ERs are present in tissues responsible for the regulation of metabolism (hypothalamus, adipose tissue, skeletal muscle, β-cells, for review see [7]), the implication that phytoestrogens regulate metabolism appears plausible. In this direction, we have recently found that CD-1 male mice exposed to high levels of dietary phytoestrogens from conception to adulthood display a reduction of adiposity [10] together with an improvement in glucose tolerance and insulin sensitivity due to an increase in glucose uptake in skeletal muscles [11]. These findings indicate that life-long exposure to dietary phytoestrogens improves metabolic functions such as adiposity and glucose homeostasis. However, little is known about the period during which cells implicated in the regulation of metabolism are sensitive to exposure to these compounds.

Numerous studies, most of which have focused on Bisphenol-A (BPA), support the hypothesis that elevated levels of natural or environmental estrogens during perinatal life may permanently affect organ development, and thus result in a predisposition to abnormal organ function or adult onset diseases [12]. It is suggested that epigenetic patterns, which are transmitted from mother to daughter cells during cell division or modified during cell differentiation when transcription of particular genes is permanently turned off or on, are irreversibly altered by the exposure to environmental compounds [13]. Identification of the time window during which an individual is more sensitive to EDCs would provide important insights into the endocrine origin of metabolic diseases. In addition, it would significantly improve fundamental understanding of the mechanisms that lead to an increased frequency of metabolic diseases.

We previously observed a decrease in adiposity and an amelioration of glucose tolerance in male mice with life-long exposure to phytoestrogens. We hypothesized that some of these beneficial effects could result from an exposure restricted to specific periods of life. Here, we found that the windows of sensitivity to phytoestrogens that lead to the improvements in adiposity and glucose tolerance do not overlap. The improvements in adiposity occur in postnatal and adult life, whereas glucose tolerance improvements are restricted to fetal exposure. In addition, we provide evidence that the intrauterine position (IUP), a model which consists of assessing in adulthood the effects of minute changes in steroid levels during fetal life, determines glucose tolerance, blood pressure and bone mass density in adult individuals.

## Results

### Phytoestrogen exposure during different developmental periods

**Figure 1:**
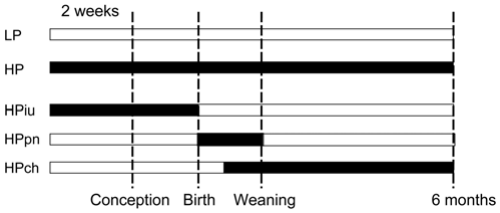
Schematic of the experimental design for period of sensitivity experiments. (A) Experimental design for *period of sensitivity* experiments. For *in utero* exposure (HPiu), male and females were fed for 2 weeks prior to mating up until birth with the HP diet, at which point the diet was replaced with the LP diet. For post-natal exposure (HPpn), female mice were fed with the LP diet until parturition, at which point the diet was shifted to the HP diet until weaning (P21). For the chronic adult exposure (HPch), animals were exposed to the HP diet only from weaning onwards. Male and female pups were normalized for litter size at birth (n = 11), with an equivalent male to female ratio.

To identify the period during which leanness or the improvement in insulin sensitivity is acquired upon exposure to dietary phytoestrogens, male mice were exposed to a soy-rich diet during specific developmental periods **(** **Figure 1A** **)**. Two groups, used as a reference, were exposed from conception onwards either to a phytoestrogen-rich diet (HP), or to a low phytoestrogen containing diet (LP). This life-long exposure to LP or HP diets encompasses the three major developmental periods (fetal, postnatal and adult), as previously published [10], [11]. Three additional groups were generated: i) *in utero* exposure (HPiu), where pregnant females consume the HP diet prior to and during the whole gestation. ii) postnatal exposure (HPpn), where lactating mothers consume the HP diet from birth until weaning so that the pups are exposed to phytoestrogens throughout lactation and iii) chronic exposure (HPch), where male pups are fed the HP diet from weaning onward.

### Fetal exposure to dietary soy improves glucose control, but not adiposity

**Figure 2:**
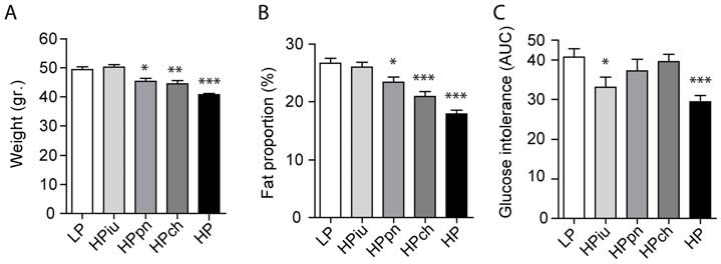
Period of sensitivity for the effects of phytoestrogen exposure on adiposity and glucose metabolism. Body weight (A) and adiposity (B) were lower only in animals exposed to the HP diet after birth. Improvements in glucose tolerance (C), represented as the mean-area-under-curve of a glucose tolerance test (GTT), was restricted to the animals fed the HP diet during fetal life (HPiu). Results are mean±SEM (n = 14-15); *p<0.05, **p<0.01, ***p<0.001 versus LP.

Different periods of exposure influenced body weight (ANOVA: *p*<0.0001) and adiposity (*p*<0.0001). HP mice showed a significant reduction in weight and adiposity when compared to LP mice as previously shown [10]. Whereas *in utero* exposure to dietary soy had no effect on body weight or adiposity, post-natal exposure was sufficient to significantly reduce weight and adiposity in comparison to life-long LP exposure **(Figure 2A, B)**. This effect was greater in chronically exposed mice (HPch), suggesting that adulthood is the most sensitive period for adipose changes triggered by dietary soy. The leanness and reduced adiposity was even more pronounced in mice exposed throughout their lives (HP mice), indicating that the post-natal and chronic effects may be additive. These results suggest that the beneficial effects of phytoestrogens on adiposity occur only after birth.

To investigate glucose tolerance, all mice were simultaneously subjected to a glucose bolus (1 mg/kg) and glycemia also appeared modulated by different periods of exposure (*p*<0.0024). For sake of clarity, glucose tolerance is represented here as an area under curve (AUC). As previously shown, HP mice displayed an improvement in glucose tolerance [11]. Interestingly, the improvement in glucose tolerance is restricted to mice exposed to dietary phytoestrogens during fetal life, whereas exposure after birth had no statistical effect on glucose tolerance despite the reduction in adiposity **(** **Figure 2C** **)**. Notably, the effects of fetal exposure to phytoestrogens did not reach the efficiency of lifelong exposure. These results suggest that the improvement in glucose homeostasis is set during fetal life, and that the beneficial effects on adiposity and insulin sensitivity are disconnected and independent.

### Fetal hormonal environment determines adult glucose homeostasis

The human fetus is very sensitive to small hormonal changes. For example, twins receive small amounts of sex hormones from their neighboring sibling during fetal development. Adult sexually dimorphic traits such as second to fourth digit finger ratio [14], auditory system [15], craniofacial growth [16], visual acuity [17], canine size [18] and reproductive fitness [19] will be influenced by the gender of the developing neighbor. In rodents, the models that enables to assess adult effects of minute changes of steroid levels during fetal life is the intrauterine position model (IUP) [20]. Due to the transfer of androgens and estrogens from adjacent fetuses [21], female or male fetuses surrounded by two males (2M) have higher amniotic or blood testosterone and lower amniotic or blood estradiol than fetuses flanked by two females (2F) [22], [23]. As a consequence 2M animals exhibit more masculinized anatomical, physiological and behavioral traits than 2F littermates (for review see [20]). Thus, variability in hormone levels in rodent and human fetuses has important “programming” consequences that can impact adult physiology and disease.

**Figure 3:**
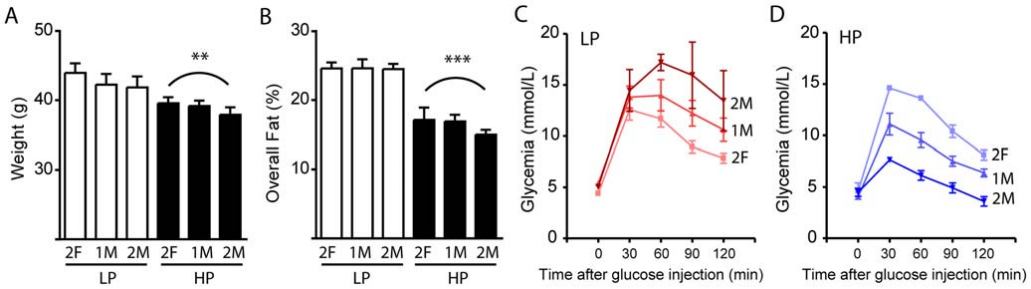
Influence of intrauterine position on adult male adiposity and glucose tolerance. Body weight (A) and fat abundance (B) were lower in HP mice versus LP mice, but intrauterine position did not influence those parameters. Glucose intolerance was higher in 2M than in 2F male mice in absence of phytoestrogens (C). Phytoestrogens drastically improved glucose tolerance in 2M male mice, but they had negligible effects in 2F male mice (D). Results are mean±SEM (n = 3-10), *p<0.05, **p<0.01, ***p<0.001 of HP groups vs LP groups.

**Figure 2** suggested that fetal hormonal environment may induce permanent changes in adult glucose homeostasis without altering adiposity in mice. To test this hypothesis, we relied on the IUP model. The uterine position of each individual was identified just before birth to allow the comparison of adult phenotypes according to their uterine environment. Animals were exposed either to the LP or HP diet throughout life. In brief, male fetuses were isolated by caesarean delivery a few hours before normal parturition, the IUP was identified (2F, 1M, 2M), the fetuses were marked so that their uterine position could be identified in adulthood, and finally eleven male and female fetuses were transferred to a foster mother. At 6 months of age, we assessed body weight and adiposity **(** **Figure 3A, B** **)**. As expected, HP mice were significantly lighter in weight and leaner than LP mice (*p* = 0.0086 and *p*<0.0001 respectively). However these parameters were not affected by the IUP since 2F, 1M and 2M mice exhibited similar body weight and adiposity relative to the rest of their group, for both the LP and HP groups. These results support our initial findings **(** **Figure 2** **)** indicating that small changes in hormonal environment during fetal development do not influence adiposity later in life.

### Fetal hormonal environment pre-determines the degree of glucose intolerance

**Figure 4:**
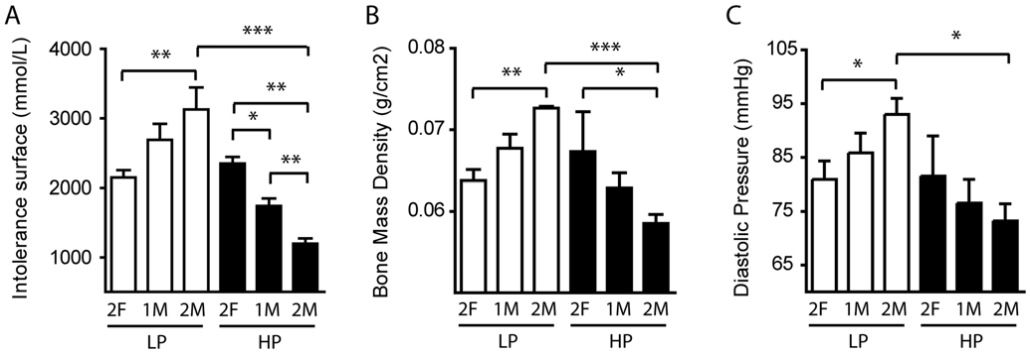
Intrauterine position affects adult bone and vascular metabolism. (A) Bar graph of the area-under-curve (intolerance surface) from the figure 3C, D. (B) Bone mass density according to the intrauterine position in LP and HP mice. (C) Influence of the intrauterine position on blood pressure in LP and HP mice. Black bars represent HP animals while empty bars symbolize LP animals. Red curves represent the GTT performed on animals fed on a LP diet, and those from HP mice are shown with blue curves. Results are mean±SEM (n = 3-10), *p<0.05, **p<0.01, ***p<0.001 between the linked groups.

Interestingly, we found that IUP affects the glycemic control of adults from both the HP and LP groups (*p* = 0.0284) **(** **Figure 3C, D** **)**. Glucose intolerance was higher in 2M LP mice when compared to 2F LP mice, suggesting that either fetal enrichment with androgens decreases glycemic control or that higher estrogen levels (here by two female embryos) improves glucose homeostasis. Phytoestrogens are considered as pseudo-agonists, since their activity depends on the level of natural estrogens. For instance, their activity is low at physiological levels of estrogen (1 nM), such as those found in pre-menopausal women, and it rises when levels of estradiol are lower (0.01 nM), such as those found in post-menopausal women [24]. Consistent with these pseudo-agonistic properties of isoflavones in presence of estrogens, exposure to phytoestrogens radically improved the glucose intolerance found in 2M males, while it had no influence in 2F males (see **Figure 4A**, which is an area under curve of Figure 3C, D). These findings suggest that the IUP affects glucose tolerance and that minute changes in estrogenic compounds can potentially trigger important changes in adult glucose tolerance.

To reinforce these findings, we measured other estrogen-related properties such as bone mass density (BMD) and blood pressure. We found that IUP influences BMD (*p* = 0.0014) with similar trends and blood pressure to a lesser extend (*p* = 0.2431), suggesting that glucose control, BMD and blood pressure may be hormonally predetermined during fetal life **(** **Figure 4A-C** **)**. Overall, it suggests that several metabolic parameters, with the exception of the regulation of adipose mass, are pre-determined during fetal life by endogenous hormones and by the exposure to dietary phytoestrogens.

## Discussion

The present findings demonstrate by two independent experiments that, in addition to the nutritional and genetic predispositions, the degree of glucose intolerance is set during fetal life by hormonal factors [i.e. environmental endocrine disrupting compounds (EEDCs) and/or endogenous steroids] independently of adipose gain. While we focused only on phytoestrogens as a natural source of endocrine disruptors, our findings raise concerns towards widespread synthetic compounds which activity can be detected as low as parts per billion doses (e.g. Bisphenol-A, a high-production-volume chemical used in the manufacture of polycarbonate plastic). *In utero* exposure to BPA at such low doses is known to cause adverse health effects such as higher body weight, increased breast and prostate cancer, and altered reproductive function. Another natural source of steroids during fetal development in humans is the maternal sex hormones. In humans, the steroid levels during pregnancy are influenced by many factors such as maternal age [25], parity [25], ethnic group [26] and associated with an increased risk of testicular germ cell tumors, prostate or breast cancer in the following generation . Further investigation is needed to evaluate the relationship between gestational exposure to EEDCs (e.g. Bisphenol-A, phtalates), maternal and fetal steroid levels and subsequent metabolic effects in adulthood.

An important question following from these findings is whether the intrauterine position has significance for humans. Even though IUP is usually considered in litter-bearing mammals where fetuses are influenced by variable levels of steroids (androgens and/or estrogens) due to the sex of neighboring fetuses, a similar situation can be found in dizygotic (DZ) twins indicating that humans are also sensitive to steroid influences by the neighboring embryo. Male-female DZ twins show higher levels of sensation seeking (the need for new and varied experiences through disinhibited behavior) than do female DZ twins [27]. Otoacoustic emissions (OAEs), which are continuous sounds produced by the cochlea, are more frequent in females than in males. Interestingly, male-female DZ twins have less OAEs than other female twins [15], [28]. Finally, breast cancer predisposition appears to be increased in female-female DZ twins when compared to male-female DZ twins [29]. Although the proportion of DZ twins within the population is on the rise, due to the increase use of *in vitro* fertilization techniques, we believe that the most relevant consequences of these findings extend to all singleton human fetuses and relate to the effects of fetal hormonal environment on adult metabolism. By hormonal environment, we mean the combination of both the endogenous estradiol/androgens ratio and EDCs. These endocrine disruptors may be dietary phytoestrogens as described here but could include other xenoestrogens, for example bisphenol A and DES.

Our findings suggest that exposure to EDCs during the fetal period could predetermine adult metabolic and cardiovascular parameters. However, the precise molecular mechanism leading to these alterations is currently unknown although epigenetic modifications of gene expression by fetal exposure to phytoestrogens are a plausible hypothesis. Several years ago, Li et al [30] demonstrated that fetal exposure to DES elicits demethylation of a single CG site in the lactoferrin promoter with persistent overexpression of the gene in mature mouse uteri. More relevant to our study, the isoflavone genistein has been shown to alter epigenetic marks in a model of adult-onset obesity [31], the yellow agouti *A^vy^* mouse, whose coat color and adiposity are dependant on the methylation state of an intracisternal A particle (IAP) retrotransposon inserted upstream of the *Agouti* gene [32], [33], [34]. In this study, Dolinoy *et al*. revealed that fetal exposure to genistein in *A^vy^* mice modified the methylation state of this IAP resulting in altered coat color and in decreased prevalence of adult obesity [31] thus demonstrating that genistein actions *in utero* can lead to methylation changes with phenotypic consequences.

Unfortunately, our experimental design presents significant technical limitations that prevent us to investigate epigenetic alterations based on the IUP and/or after exposure to dietary phytoestrogen during the fetal period. This is mainly due to a combination of parameters including the low doses of fairly weak estrogenic chemicals coupled with subtle, late-onset multifactorial phenotypes that are rarely fully penetrant. In addition, the use of an outbred strain precludes epigenetic analyses. Assessing the intra-uterine position (IUP) in mice requires females capable of producing large litter size which is only possible with outbred strains (e.g. CD-1). This need is linked with technical and experimental reasons: the IUP is determined after caesarean delivery a few hours prior birth, and pups have then to be transferred to a foster mother. More importantly, the two pups located at both extremities of each uterine horn are eliminated and only male pups in between are selected for IUP analysis. Thus, small litter size, such as that of inbred strains, drastically reduces the chances of obtaining 2F or 2M mice. To circumvent the limitations of genetic variability, we attempted numerous times to recapitulate our IUP analyses with a C57/B6 inbred strain but failed, simply due to the difficulty of obtaining enough 2M and 2F male mice for subsequent metabolic analysis. Finally, identifying the epigenetic modulations caused by the fetal exposure to dietary phytoestrogens or the IUP would require first to identify which organs/tissues are responsible for the changes in glucose tolerance without changes in lipid abundance through physiological and molecular characterizations. Only then, a thorough epigenetic analysis which includes whole-epigenome profiles combined with transcriptome analyses could be undertaken.

Very few studies have evaluated the effects on metabolism of *in utero* exposure to environmental compounds. Usually, these studies are restricted to glucocorticoid overexposure (i.e. dexamethasone, or due to maternal stress) during gestation and intrauterine growth retardation, both known to program adult onset disorders such as cardiovascular diseases (hypertension), and metabolic (i.e. hyperglycemia, hyperinsulinemia), endocrine and behavioral disorders [35], [36]. For instance, maternal stress during gestation leads to adult glucose intolerance [37]. The placental 11β-hydroxysteroid dehydrogenase type 2 is known to protect the fetus from maternal glucocorticoids. Inhibition of this enzyme during fetal life of rats leads to a decrease in body weight and glucose intolerance in adulthood [38], an effect that is restricted to late gestation [39]. Thus, interference with the function of this enzyme and glucocorticoid sensitivity might lead to increase risk of developing adult diseases. A rise of testosterone levels during fetal life is observed after maternal stress, suggesting that fetal androgens may also be implicated in such diseases. Although the levels of testosterone were not measured in the studies mentioned here, several reports using the IUP model have shown that pregnant mice subjected to intense light and heat during the final third of gestation have increased serum corticosterone levels [40]. This leads to an increase in testosterone levels in 2F male fetuses such that that they behaved as 2M males in adulthood [41], [42]. Why 2F males are more sensitive to glucocorticoids is still unknown. Nonetheless, 2M males exhibit behaviors that resemble those of 2F exposed to glucocorticoids. Overall, these studies are consistent with the present work, showing that 2M males are more prone to develop diabetes and hypertension than are 2F males.

The effects observed upon exposure to dietary phytoestrogens are consistent with the described pseudo-agonistic properties of these compounds. The effects on glucose tolerance, bone mass density and hypertension are greater in 2M males, were estradiol levels are lower than in 2F males, leading to impressive changes. This raises questions as to whether the effects of IUP are due to either estrogenic (or anti-androgenic) or androgenic (or anti-estrogenic) actions. The complexity of the dual roles played by both estrogens and androgens is illustrated by studies in mouse models lacking either the androgen receptor (ARKO) or the estrogen receptor alpha (ERαKO). While one would expect differing phenotypes, increases in weight gain and adiposity, glucose intolerance, and insulin insensitivity, are observed in both AR deficient and ERα deficient mice [43], [44], [45], [46]. Adult phenotypes resulting from specific intrauterine positions should thus be regarded as complex estrogen-androgen interactions.

Bone mass density is tightly regulated by both male and female hormones [47]. The decrease in bone mass density observed in adult 2F LP males or in 2M HP males may indicate a predisposition to osteoporosis due to the presence of fetal estrogens. In females, estrogen is required for the modeling of trabecular bones via ERα [48]. In contrast, studies using androgen receptor and estrogen receptor alpha double knock-out mice (AR-ERαKO) have shown that androgen, but not estrogen, actions are required for the modeling of trabecular bone in males. Conversely, the modeling of cortical bone requires the additive effects of both androgens and estrogens [47]. Further studies will be required to elucidate whether the microarchitecture, such as trabecular or cortical bone volume and thickness, and the stiffness are affected in order to understand the hormonal origins of the decreased BMD in 2F males. It is interesting to note that genetic factors influence bone mass density in humans as much as 85% [49]. Recently, epidemiological evidence has suggested that there are correlations between fetal and post-natal life, and fracture risk in adulthood [50], [51]. In most cases, the fetal origins of osteoporosis are due to undernourishment during embryonic life [52]. Our observations suggest that, in addition to nutritive factors and genetics, adult BMD can also be influenced by fetal endocrine cues.

Studying EDCs is a difficult task. For practical purposes, we used CD-1 mice due of their excellent reproductive characteristics (11.5 pups per litter). Unfortunately, because of the mixed genetic background of CD-1 mice, genetic and epigenetic analyses are precluded. However, this model may prove useful in studying the consequence of multiple ED exposure *in utero* on adult metabolic and cardiovascular parameters. The IUP may serve as a tool to directly influence sensitivity to a mixture of EDs, and would therefore allow the assessment of whether these exogenous molecules act in an additive or synergistic fashion to modulate adult metabolic parameters.

## Materials and Methods

### Diets and animal care

CD-1 mice had *ad libitum* access to either a high soy-containing [high phytoestrogen (HP)] diet (Harlan Teklad 8604; Harlan Teklad, Madison, WI, USA], or a soy-free [low phytoestrogen (LP)] diet (Zeigler Phytoestrogen Reduced Rodent Diet I; Zeigler Brothers, Gardner, PA, USA). The isoflavone content of these two closed-formula diets is approximately 355 ppm daidzein and 389 ppm genistein equivalents in the HP diet and nondetectable in the LP diet (Analysis performed by Lareal, Vannes, France). These concentrations of isoflavones are consistent with a soy protein content of approximately 25% in the HP diet. Animals fed with the HP diet had serum isoflavone levels of 0.3 µM (genistein or daidzein) to 8 µM (equol), whereas in LP mice, the levels were barely detectable [10], [11]. Both diets are equivalent in terms of carbohydrate, protein, fat, amino acid, vitamin, and mineral content [53]. In the LP diet formulation, soy was omitted and replaced by lactic casein and dried skim milk. The gross energy content (1,626 mJ/100 g for the HP diet versus 1,668 mJ/100 g for the LP diet), the metabolizable energy (3,100 vs. 3,240 kcal/kg), and the digestable energy (3,300 vs 3,530 kcal/kg) were similar for both diets. CD-1 male and female mice were purchased from Charles River (Arbresle, France) for breeding. Animal protocols used in these studies were approved by the Commission d'Ethique de l'Expérimentation Animale of the University of Geneva Medical School and the Geneva Veterinarian Office. Animals were treated humanely and with regard for the alleviation of suffering. Mice were housed in polystyrene instead of polycarbonate cages to avoid potential contamination with bisphenol-A, a well-known endocrine-disrupting compound with estrogenic activity.

### Body composition determination

Peripheral dual energy x-ray absorptiometry (pDXA; PIXImus, GE-Lunar Corp., Madison, WI, USA) was used to measure *in vivo* percent fat mass of mice as previously described [10].

### Glucose tolerance test

For glucose tolerance tests (GTTs), animals fasted overnight (11 h) were injected intraperitoneally with 1.5 g glucose/kg body wt. Plasma glucose levels were measured at 0, 30, 60, 90, and 120 min with Glucometer DEX (Bayer) as described in [11].

### Blood pressure measurements

Tail cuff systolic blood pressure was measured in mice using a noninvasive computerized tail cuff system (BP-2000, VisiTech Systems, Apex, NC; [54], [55], [56]. Mice were trained for 1 week, and then systolic and diastolic blood pressures were measured as the mean of at least 15 to 20 successful measurements.

### Statistical analysis

Results are expressed as means±SE of *n* experiments. ANOVA or the nonparametric unpaired *t* test was used for statistical analysis when appropriate. Differences were considered statistically significant if *P* was <0.05.
